# High Hemocyte Load Is Associated with Increased Resistance against Parasitoids in *Drosophila suzukii*, a Relative of *D. melanogaster*

**DOI:** 10.1371/journal.pone.0034721

**Published:** 2012-04-17

**Authors:** Balint Z. Kacsoh, Todd A. Schlenke

**Affiliations:** Biology Department, Emory University, Atlanta, Georgia, United States of America; American Museum of Natural History, United States of America

## Abstract

Among the most common parasites of Drosophila in nature are parasitoid wasps, which lay their eggs in fly larvae and pupae. *D. melanogaster* larvae can mount a cellular immune response against wasp eggs, but female wasps inject venom along with their eggs to block this immune response. Genetic variation in flies for immune resistance against wasps and genetic variation in wasps for virulence against flies largely determines the outcome of any fly-wasp interaction. Interestingly, up to 90% of the variation in fly resistance against wasp parasitism has been linked to a very simple mechanism: flies with increased constitutive blood cell (hemocyte) production are more resistant. However, this relationship has not been tested for Drosophila hosts outside of the melanogaster subgroup, nor has it been tested across a diversity of parasitoid wasp species and strains. We compared hemocyte levels in two fly species from different subgroups, *D. melanogaster* and *D. suzukii*, and found that *D. suzukii* constitutively produces up to five times more hemocytes than *D. melanogaster*. Using a panel of 24 parasitoid wasp strains representing fifteen species, four families, and multiple virulence strategies, we found that *D. suzukii* was significantly more resistant to wasp parasitism than *D. melanogaster*. Thus, our data suggest that the relationship between hemocyte production and wasp resistance is general. However, at least one sympatric wasp species was a highly successful infector of *D. suzukii*, suggesting specialists can overcome the general resistance afforded to hosts by excessive hemocyte production. Given that *D. suzukii* is an emerging agricultural pest, identification of the few parasitoid wasps that successfully infect *D. suzukii* may have value for biocontrol.

## Introduction

Fruitflies of the genus Drosophila are regularly attacked by parasitoid wasps. In natural *D. melanogaster* populations, upwards of 50% of fly larvae are found to be infected by wasps, suggesting they exert extremely strong selection pressures on Drosophila populations in nature [1], [2], [3]. Once infected, fruitfly larvae mount an immune response against wasp eggs, termed melanotic encapsulation, that is thought to involve several steps [4], [5]: The response begins when circulating, constitutively produced plasmatocytes recognize the wasp egg as foreign and signal to induce the differentiation of larger lamellocytes from pro-hemocytes in the lymph gland (the fly hematopoietic organ) and from other circulating plasmatocytes (via the intermediate podocyte form) [6], [7]. These newly derived lamellocytes migrate towards, and attach and spread around the wasp egg in a multi-layered capsule. In the final step, the inner cells of the capsule surrounding the wasp egg lyse and release reactive oxygen species and an impermeable layer of melanin, resulting in death of the wasp egg. However, parasitoid wasps can potentially evade host immune responses by using a non-reactive coating on their eggs, or suppress host immunity by injecting venom into hosts along with their eggs. There is both between and within species genetic variation in flies for resistance against wasps and among wasps for virulence against flies [8], [9], [10], [11], [12], [13], [14], [15], [16].

In previous work, Drosophila species from the melanogaster subgroup were found to have significantly different numbers of constitutively produced plasmatocytes, and there was a significant correlation (r^2^ = 0.90) between plasmatocyte counts and ability to melanotically encapsulate the eggs of the immune-evasive parasitoid wasp *Asobara tabida* [12]. It was also found that *D. melanogaster* strains artificially selected for resistance against *A. tabida* showed a significant increase in plasmatocyte numbers [17]. Furthermore, *D. simulans*, which makes significantly more plasmatocytes than its sister species *D. melanogaster*, was significantly more resistant against the more immune-suppressive wasp *A. citri* [18]. Finally, *D. melanogaster* mutants producing a wide range of hemocyte counts showed a significant correlation (r^2^ = 0.45) between constitutive plasmatocyte numbers and encapsulation ability against the wasp *Leptopilina boulardi* [19]. Altogether, this work suggests that high constitutive production of hemocytes is an effective and relatively simple mechanism by which hosts can evolve resistance to one of their most common groups of parasites.

We were interested in whether the relationship between Drosophila standing immune defense (hemocyte production) and immune resistance against wasps is general across a large panel of diverse wasp lineages with unique infection strategies, and whether the relationship extends beyond the melanogaster subgroup of the genus Drosophila. Pilot data from a study aimed at characterizing hemocyte lineages across the genus Drosophila (unpublished) suggested *D. suzukii*, a member of the melanogaster group but not the melanogaster subgroup, constitutively produces an extremely large number of hemocytes compared to other Drosophila. Thus, the goal of this study was to confirm whether *D. suzukii* constitutively produces higher numbers of hemocytes than *D. melanogaster*, and if so, to determine whether *D. suzukii* was also more resistant against a large panel of parasitoid wasp species and strains.

**Figure 1:**
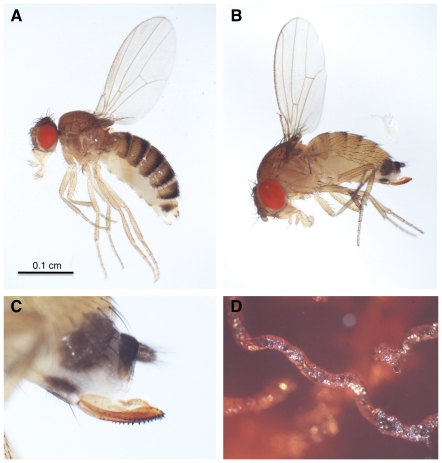
Fly morphology and behavior. (A) Female *D. melanogaster*; (B) female *D. suzukii*; (C) serrated ovipositor from female *D. suzukii*; (D) tunnel excavated by *D. suzukii* larva through agar food plate.

*D. suzukii* is native to east Asia but has recently gained widespread attention due to its spread as an agricultural pest in Europe and North America (Figure 1) [20], [21], [22], [23]. Although most of the ∼1,500 described Drosophila species lay their eggs and feed on decaying plant and fungal tissues, including rotting fruits (like *D. melanogaster*), *D. suzukii* is one of a handful of species that live on ripe fruits, using its serrated ovipositor to lay eggs in the flesh of soft-skinned fruits (Figure 1C). Its larvae subsequently burrow through the body of the fruit as they eat (Figure 1D), allowing bacteria and other microorganisms access to the inside of the fruit, which results in premature rotting. Because parasitoid wasps have been successfully used as biocontrol agents against a wide range of insect agricultural pests, including Coleopterans (*e.g.*, weevils, bean beetles), Hemipterans (*e.g.*, scale insects, whiteflies, aphids, leafhoppers, stinkbugs), Lepidopterans (*e.g.*, various moth and butterfly larvae), and Dipterans (*e.g.*, Tephritid fruitflies, blackflies) [24], [25], [26], [27], [28], study of *D. suzukii* resistance and susceptibility to parasitoid wasps may have added applied value.

At least four families of parasitoid wasps are known to attack Drosophila in nature [29]. These wasps use a variety of infection strategies to defeat the fly immune response, including immune suppressive and evasive tactics, and vary in their host ranges from specialists of particular Drosophila species to generalist of the genus. Members of the families Braconidae and Figitidae are larval parasites – they lay single eggs in Drosophila larvae and, if not killed, the hatched wasp larva begins to consume internal fly tissues before eventually killing the fly and eclosing from the fly pupal case. Members of the families Diapriidae and Pteromalidae are pupal parasites - they lay single eggs inside Drosophila pupae, and the hatched wasp larva consumes the fly pupal tissues, also eventually killing the fly and eclosing from the fly pupal case. It is unclear whether fly pupae can mount an immune response or otherwise defend themselves once infected by pupal parasites. Pupal parasites of the genus Trichopria (Family Diapriidae) lay their eggs in the Drosophila hemocoel, like larval parasites, but those of the genus Pachycrepoideus (Family Pteromalidae) lay their eggs in the space between the Drosophila pupal case and the pupa, and act as ectoparasites in the early stages of their life by sucking fluids from the pupa externally [29]. A lack of pupal immunity against wasps may explain in part why pupal parasitoid wasps are thought to have more generalist host ranges than larval parasitoid wasps [30], [31].

The Drosophila-wasp system is ripe for study as a model for the co-evolution of pathogen infection strategies and host immune responses across lineages and communities of pathogens and hosts [32]. We attempted to answer the following questions: Is the melanotic encapsulation response observed in *D. melanogaster* conserved in *D. suzukii*? Does *D. suzukii* have higher constitutive hemocyte production than *D. melanogaster*? Is increased hemocyte production by *D. suzukii* associated with stronger resistance against a panel of parasitoid wasps with diverse life histories and infection strategies? Do wasps make different oviposition choices depending on host species? Do wasp phylogeny and biogeography play any role in fly-wasp interactions? From an applied point of view, which parasitoid wasp species show the most potential for use in *D. suzukii* biocontrol in the field?

## Materials and Methods

### Insect Species

The *D. melanogaster* genome strain 14021-0231.36 was acquired from the Drosophila Species Stock Center and was grown on standard cornmeal/yeast/molasses Drosophila medium. The two additional *D. melanogaster* strains originated from single wild-caught females collected in Atlanta, GA in the summer of 2010. The primary *D. suzukii* strain tested originated from four wild-caught females collected in Atlanta, GA in the summer of 2010, while two additional isofemale strains were collected in Atlanta, GA in the summer of 2011. *D. suzukii* were maintained on standard Drosophila medium supplemented with (thawed) frozen raspberries, which were found to enhance egg-laying but were otherwise unnecessary for fly development.

**Figure 2:**
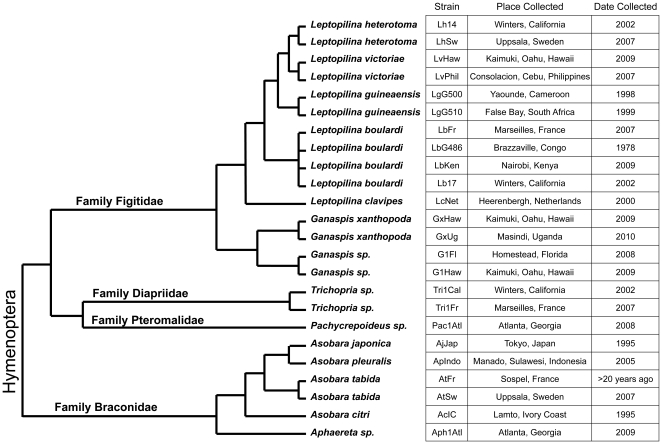
Phylogenetic relationships and provenance of wasps used in this study. Tree topology is derived from previous phylogenetic studies of Hymenopteran families [68], the family Figitidae [69], [70], and the family Braconidae [71]. Branch lengths are approximated.

A total of 24 Drosophila parasitoid wasp strains collected from around the world were used for infection trials on *D. melanogaster* and *D. suzukii* (Figure 2). Strains LgG500 and LgG510 were provided by R. Allemand, strain LbG486 was provided by D. Hultmark, strains LcNet, AjJap, ApIndo, and AcIC were provided by J. van Alphen, strain GxUg was provided by J. Pool, and strain AtFr was provided by B. Wertheim. All other strains were collected by the Schlenke lab. These wasp strains represent: (1) at least 14 species, (2) representatives of all four Hymenopteran families known to infect Drosophila, (3) larval and pupal parasites, and (4) a worldwide range of collection localities (Figure 2). Morphology and *cytochrome oxidase I* (*COI*) sequences from the two *Trichopria sp.1* strains suggested they were representatives of the same species, perhaps *Trichopria drosophila* (Ashmead). Furthermore, morphology and *COI* sequences from the two *Ganaspis sp.1* strains suggest they are representatives of a single undescribed species. All wasp species were maintained in the lab on *D. melanogaster* strain Canton S, with the exception of *L. clavipes*, *A. tabida*, *Aphaereta sp.1*, and *Pachycrepoideus sp.1*, which were maintained on *D. virilis*. To grow wasps, adult flies were allowed to lay eggs in standard Drosophila medium for several days before they were replaced by adult wasps, which then attacked the developing fly larvae or pupae. Wasp vials were supplemented with approximately 500 uL of a 50% honey/water solution applied to the inside of the cotton vial plugs. *COI* sequences for all wasp strains as well as ITS2 sequences for Figitid wasps have been deposited in Genbank under accession numbers JQ808406–JQ808451. Wasp strains are available upon request.

### Hemocyte Counts

Fly-wasp development for all experiments took place in a 25°C incubator on a 12∶12 light∶dark cycle. For hemocyte count experiments, adult female *D. melanogaster* and *D. suzukii* were allowed to lay eggs into fly food supplemented with yeast paste (50∶50 mix of baker's yeast and water) or raspberries, respectively, in 60 mm Petri dishes. After 72 hours, adult flies were removed and developmental stage and size-matched second instar fly larvae were collected for two independent experiments.

For hemocyte count experiments, *D. melanogaster* and *D. suzukii* larvae were either uninfected or were infected by the wasp strain LbG486, with three replicates per treatment. For parasitoid infections, 50 fly larvae were moved into 35 mm diameter Petri dishes filled with 1 mL of Drosophila medium. Ten female wasps were immediately allowed to attack these fly larvae for 3 hours, and five larvae per dish were later dissected to determine the number of wasp eggs laid per fly larva. Fourteen of fifteen *D. melanogaster* larvae across the three replicates were found to be infected by single wasp eggs, as well as fourteen of fifteen *D. suzukii* larvae, so we assumed the wasp infection rate was very similar across the two host fly species. Hemocytes were counted at two time-points, 12 and 24 hours post-infection, in which the induced cellular immune response was expected to be highly activated. Crystal cells, a distinct hemocyte type described below, were counted independently 33 hours post-infection.

In an experiment to test hemocyte induction absent wasp venom effects, *D. melanogaster* and *D. suzukii* larvae were either untreated or were pierced with a sterile needle to simulate the wounding associated with wasp oviposition. Such wounding has been shown to induce the production of lamellocytes [33]. For each of four replicates, 15 fly larvae were rinsed in 1× PBS, dried on Kimwipes, and immobilized on double sided tape. Their posterior cuticles were then pierced with flame-sterilized 0.1 mm diameter stainless steel dissecting pins (Fine Science Tools 26002-10), with care taken to avoid harming internal organs. Fly larvae were then removed from the tape with a wet paintbrush, and allowed to recover in a moist chamber for one hour before being moved to 35 mm diameter Petri dishes filled with 1 mL of Drosophila medium. Control larvae were treated identically except without piercing. Hemocytes were then counted 24 hours post-infection, while crystal cells were counted independently 33 hours post-infection.

**Figure 3:**
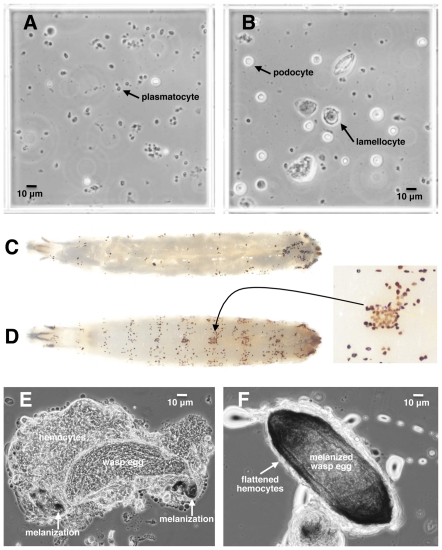
*D. suzukii* hemocytes and encapsulation of wasp eggs. (A) A 0.25×0.25×0.1 mm hemocytometer field from normal *D. suzukii* larvae showing abundant plasmatocytes; (B) hemocytometer field from *D. suzukii* larvae 12 hours after infection by wasp strain LbG486 showing increased podocyte and lamellocyte numbers; (C) control *D. melanogaster* larva with melanized crystal cells; (D) control *D. suzukii* larva with melanized crystal cells, showing color variation in inset; (E) initiation of encapsulation of LbG486 egg by *D. suzukii* showing loose hemocyte aggregation and melanization at anterior and posterior tips of egg; (F) LbG486 egg melanotically encapsulated by *D. suzukii*, showing surrounding layer of tightly spread hemocytes.

To count hemocytes, 5 third instar larvae from each treatment replicate (including controls) were washed in Drosophila Ringer's solution, dried on a Kimwipe, and bled together into 20 µL of 1× PBS solution containing 0.01% phenylthiourea on a glass slide. Dissection into buffer limits evaporation, and phenylthiourea prevents the hemolymph from melanizing [34]. The buffer-hemolymph mixture was applied to a disposable hemocytometer (Incyto C-Chip DHC-N01) and allowed to sit for 30 minutes to allow hemocytes to settle. Hemocytes from each sample were counted from sixteen 0.25×0.25×0.1 mm squares (*e.g.*, Figure 3A, 3B), which make up a total volume of 0.1 µL. Thus, the number of hemocytes from the whole 20 µL sample is expected to be ∼200 times the number counted, or a per larva value of 40 times the number counted.

The addition of hemolymph to the 20 µL of buffer is expected to increase the total buffer-hemolymph volume to greater than 20 µL, leading to a downward bias in our absolute hemocyte counts. However, the amount of hemolymph from five third instar larvae is only approximately 2.5 µL, and in practice about this much liquid evaporates before 20 µL of the buffer-hemolymph mixture can be pipetted onto the hemocytometer. Our hemocyte counts may also underestimate true hemocyte loads because a large fraction of plasmatocytes are sessile (*i.e.*, docked on host tissues) [35], and may not detach from the larval tissues upon dissection. *D. melanogaster* and *D. suzukii* adults and larvae are similar in size, (Figure 1, 3), so we did not expect differences in species hemocyte counts to result from fly size differences, but we were careful to use larvae of the same size and developmental stage from both species for all experiments. Hemocytes were classified as plasmatocytes (small round cells with obvious nuclei), podocytes (activated plasmatocytes that are larger and refract more light than plasmatocytes), and lamellocytes (large, clear flattened cells) [7].

**Figure 4:**
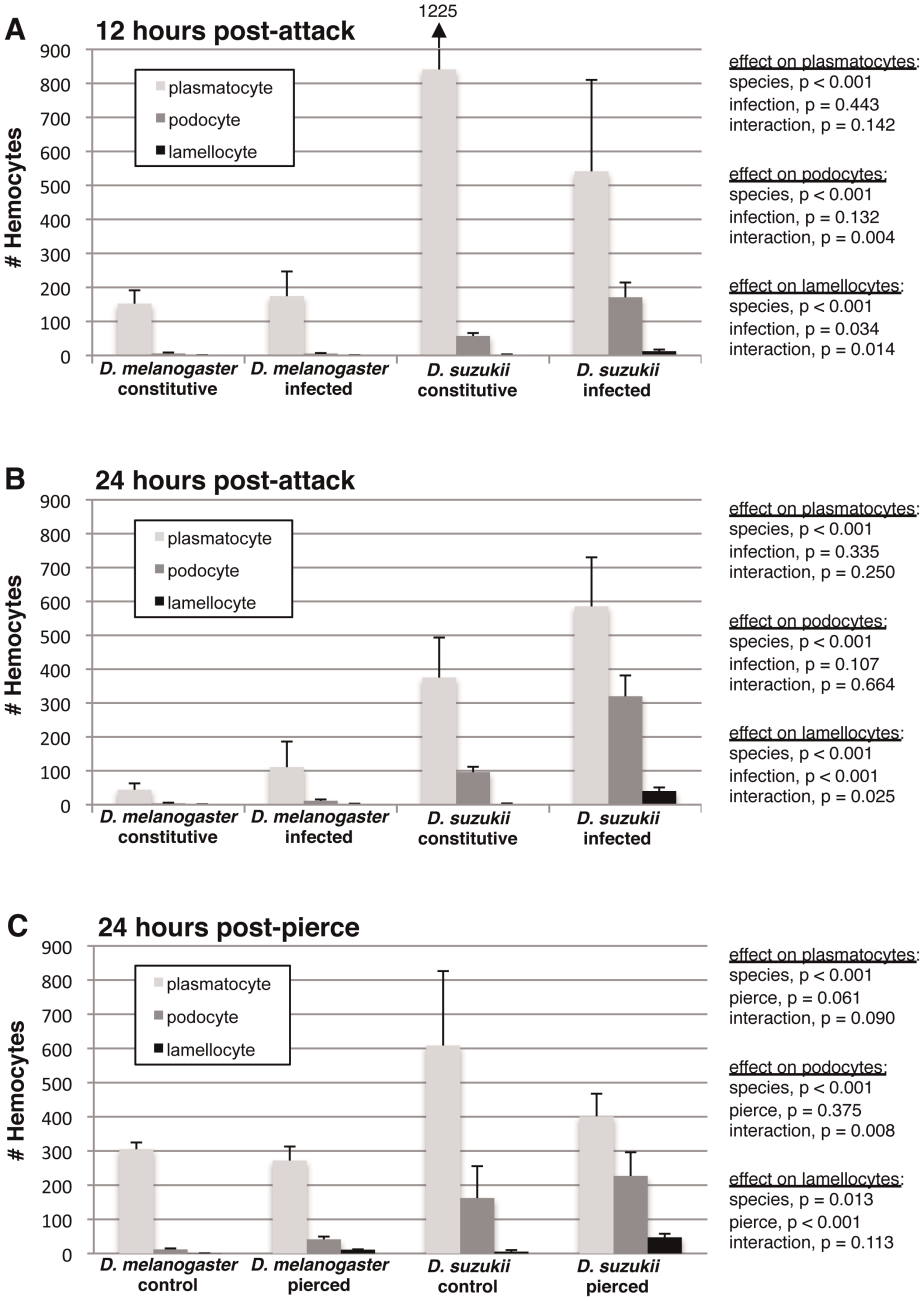
Hemocyte count comparison between *D. melanogaster* and *D. suzukii*. (A) 12 hours after infection by wasp strain LbG486; (B) 24 hours after infection by wasp strain LbG486; (C) 24 hours after piercing with a sterile needle. Average (+) standard deviation shown. Numbers are approximately one fortieth of the number of cells per one fly larva (Methods).

The fourth hemocyte cell type, crystal cells, are medium sized cells containing cytoplasmic crystals made up of the substrate that the phenoloxidase enzymatic cascade converts into melanin [36]. The crystals are rapidly lost upon dissection and the cells become difficult to recognize, so a separate method was used to count them. Crystal cells self-melanize when larvae are incubated at 60°C for 10 minutes [37]. Therefore, crystal cells were quantified separately by counting dark spots from the dorsal side of incubated whole larvae (*e.g.*, Figure 3C, 3D) at 33 hours post-infection. Crystal cells were counted and averaged from three larvae per replicate. It is not yet known whether crystal cells play a role in the melanotic encapsulation response [4].

**Figure 5:**
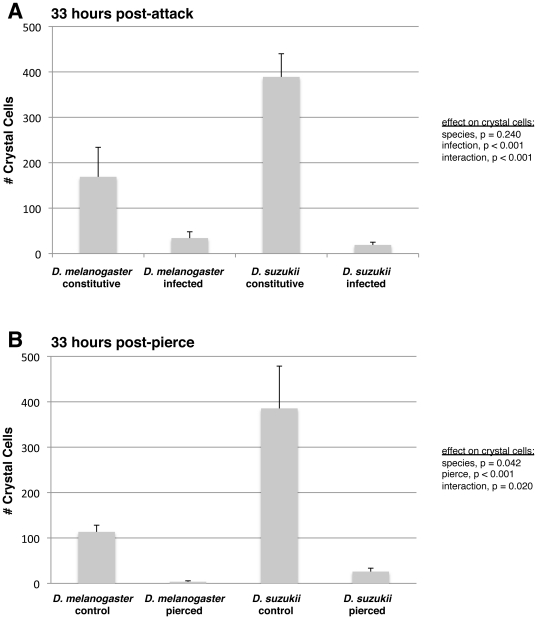
Crystal cell count comparison between *D. melanogaster* and *D. suzukii*. (A) 33 hours after infection by wasp strain LbG486; (B) 33 hours after piercing with a sterile needle. Average (+) standard deviation shown.

Multivariable regression models assuming Poisson distributions were specified to model hemocyte counts by fly species and immune challenge (wasp infection, piercing). When hemocyte counts were overdispersed, negative binomial distributions were specified instead of Poisson distributions.

### Resistance Trials

Each fly-wasp infection combination was replicated three times. Adult female *D. melanogaster* and *D. suzukii* were allowed to lay eggs into fly food supplemented with yeast paste (50∶50 mix of baker's yeast and water) or raspberries, respectively, in 60 mm Petri dishes. After 72 hours, adult flies were removed and size-matched second instar fly larvae were collected for infections. For larval parasitoid infections, 50 fly larvae were moved into 35 mm diameter Petri dishes filled with 1 mL of Drosophila medium. Three female wasps were immediately allowed to attack these fly larvae for 72 hours. After attack, 10 of the 50 fly larvae were dissected to determine the percent of larvae infected, the number of wasp eggs laid per fly larva, and the proportion of fly larvae bearing encapsulated wasp eggs in each sample. 30 of the 40 remaining larvae were then moved into Drosophila vials to complete development. For pupal parasitoid infections, 40 fly larvae were moved into vials containing Drosophila medium, and were allowed to develop another 72 hours to the wandering third instar stage, just before they began pupating on top of the medium or on the sides of the vials. Three female wasps were then allowed to attack the fly pupae for 72 hours, at which time the wasps were removed and the fly pupae were left to complete development. The infection conditions were chosen to be optimal for wasp success. Control uninfected flies from both species were reared under identical conditions and showed nearly 100% survival (data not shown).

**Figure 6:**
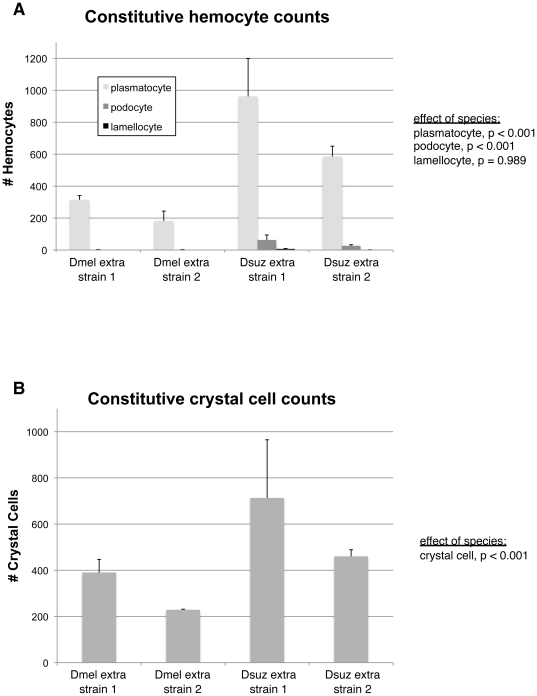
Hemocyte counts in other *D. melanogaster* and *D. suzukii* strains. (A) Constitutive plasmatocyte, podocyte, lamellocyte counts; (B) constitutive crystal cell counts. Average (+) standard deviation shown.

The total numbers of flies and wasps that eclosed from all wasp treatments were determined 15 days and 30 days post-infection, respectively, times by which all viable flies and wasps should have emerged. Fly-wasp interactions may yield one of three outcomes, which were compared between *D. melanogaster* and *D. suzukii* infections: (1) a successful immune response by the fly, (2) a successful parasitism by the wasp, or (3) death of the fly and the wasp within it. Furthermore, for larval parasitoid infections, the numbers of wasp eggs counted from dissected fly larvae were assessed for evidence of under-dispersion, as wasps are known to preferentially choose un-infected hosts for oviposition [38], [39], [40], [41]. If wasps layed eggs in fly larvae randomly, without regard to host infection status, the number of wasp eggs per larva would have been expected to follow a Poisson distribution, where the average number of wasp eggs per fly larva and the variance in the number of wasp eggs per fly larva should have been equal. Thus, for each fly-wasp pair, we compared the average number of wasp eggs laid per 10 dissected fly larvae to the variance in the number wasp eggs laid per 10 dissected fly larvae across the three replicates of each treatment, using one-tailed paired t-tests. Although some figures show data for each wasp strain separately, values for wasp strains of the same species were averaged into single species values for all statistical analyses unless otherwise noted.

**Figure 7:**
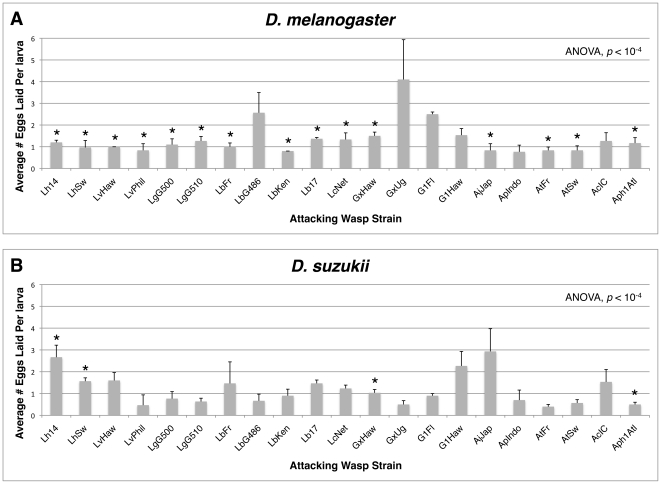
Numbers of eggs laid by each wasp strain in *D. melanogaster* (A) and *D. suzukii* (B). Average number of eggs per larva (+) standard deviation shown. ANOVA results compare egglay numbers within fly species across wasp treatments. * = significant under-dispersion of wasp eggs in fly larvae at *p*<0.05 using a one-tailed paired t-test (Methods).

**Figure 8:**
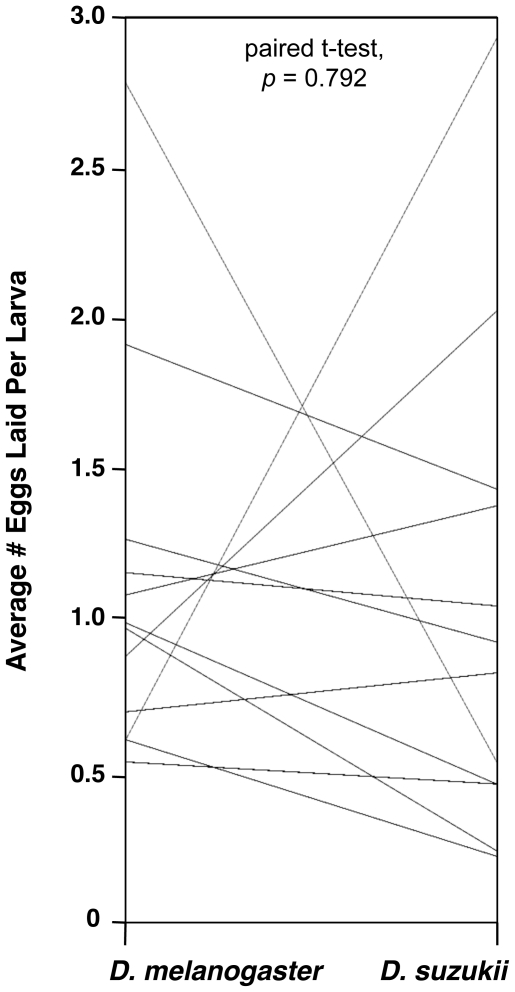
Parallel plot comparing average egglay numbers for each wasp species between hosts. There was no overall difference between fly species in numbers of eggs laid by wasps, nor was there a correlation between the number of eggs laid in *D. melanogaster* and the number of eggs laid in *D. suzukii* across the panel of wasp species (as indicated by the non-parallel connecting lines).

## Results

### Hemocytes

*D. suzukii* hemocytes were morphologically similar to those of *D. melanogaster* (Figure 3). In normal *D. suzukii* larvae, there were an abundance of small round cells in the hemolymph that were presumably homologous to plasmatocytes. In *D. suzukii* infected by wasps, medium-sized round cells resembling podocytes became much more numerous, as well as large irregular shaped cells that resembled *D. melanogaster* lamellocytes. Heating *D. suzukii* larvae resulted in the formation of darkened cells throughout the hemocoel. In *D. melanogaster,* this phenomenon has been attributed to the self-melanization of crystal cells, and suggested that *D. suzukii* also possesses hemocytes responsible for carrying melanization factors. Interestingly, while all self-melanized crystal cells in *D. melanogaster* were dark black (Figure 3C), *D. suzukii* showed both brown and black cells (Figure 3D, inset). Finally, *D. suzukii* larvae encapsulated and melanized wasp eggs with hundreds of hemocytes that flattened and spread over the wasp eggs to form a tight capsule (Figure 3E, 3F). Thus, the stereotypic melanotic encapsulation response used by *D. melanogaster* against parasitoid wasps appears to be conserved in its relative, *D. suzukii*.

**Figure 9:**
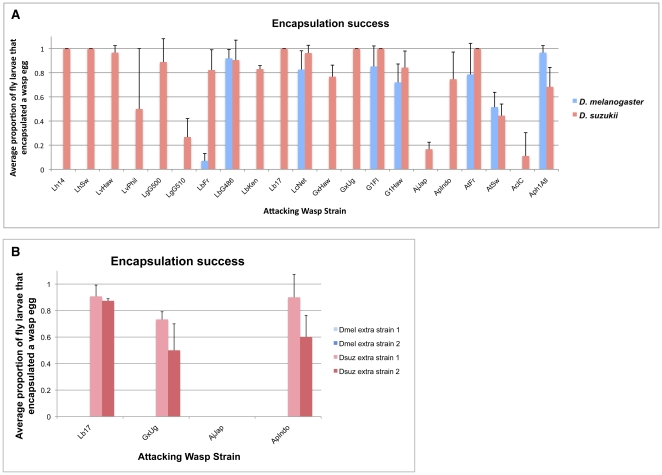
Encapsulation success of wasp-infected fly larvae. (A) Average proportion of fly larvae that encapsulated a wasp egg; (B) average proportion of fly larvae from additional fly strains that encapsulated a wasp egg.

Though hemocyte morphology was similar in the two fly species, we found significant differences in constitutive and induced hemocyte counts between *D. melanogaster* and *D. suzukii*. We used two methods for inducing immune responses in these flies. First, we infected flies with wasp strain LbG486, which is relatively avirulent in *D. melanogaster* and has been shown to induce production of hemocytes, and especially lamellocytes, in particular infected *D. melanogaster* strains [42], [43]. Second, in order to stimulate lamellocyte production in the absence of any possible immune inhibitory effects of wasp venoms, we pierced *D. melanogaster* and *D. suzukii* larvae with sterile needles [33].

**Figure 10:**
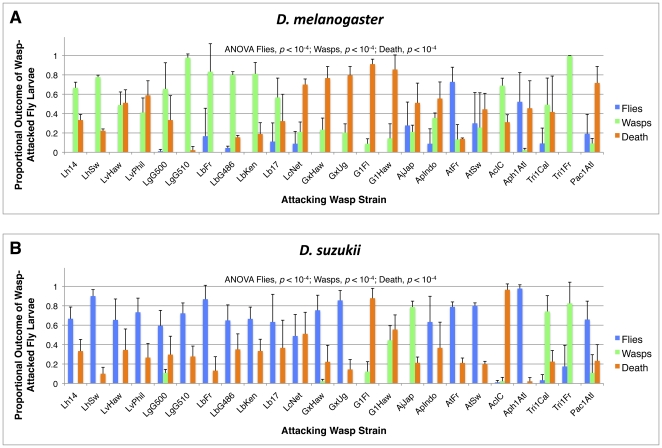
Infection outcomes for host larvae infected by each wasp strain. Average (+) standard deviation shown for *D. melanogaster* (A) and *D. suzukii* (B). ANOVA results compare fly eclosion, wasp eclosion, or death proportions within fly species across wasp treatments.

**Figure 11:**
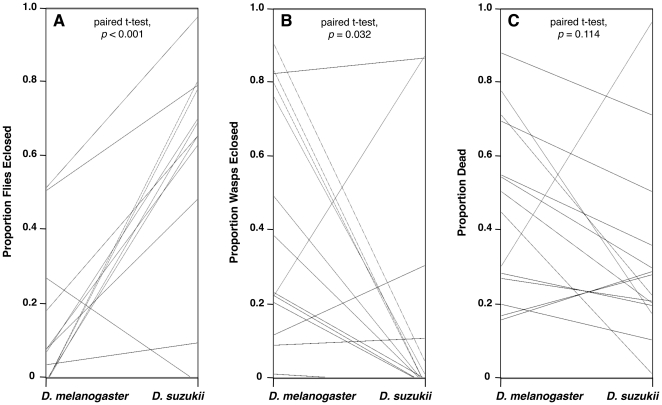
Parallel plot comparing outcomes between host larvae infected by each wasp species. (A) fly eclosion; (B) wasp eclosion; (C) death. There were significant overall differences between fly species in fly eclosion and wasp eclosion proportions, but not in proportion dead. There is no correlation between fly eclosion, wasp eclosion, or death proportions between *D. melanogaster* and *D. suzukii* across the panel of wasp species (as indicated by the non-parallel connecting lines).

**Figure 12:**
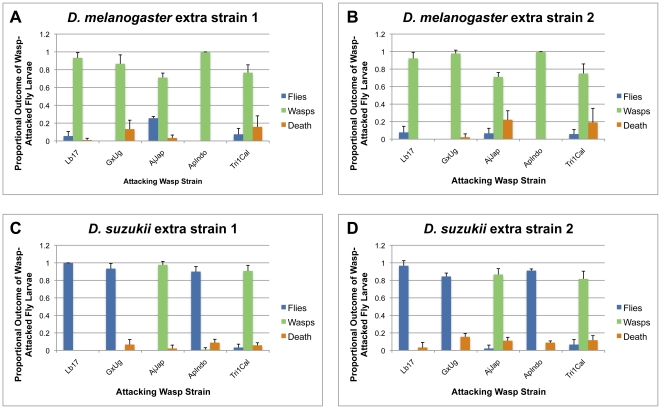
Infection outcomes for host larvae of other strains. (A, B) *D. melanogaster* extra strain 1 and 2; (C, D) *D. suzukii* extra strain 1 and 2. Average (+) standard deviation shown.

We tested the effects of fly species and immune challenge on fly hemocyte counts using standard regression methods (Figure 4). We found consistent, significant species effects on plasmatocyte, podocyte, and lamellocyte numbers. Across time-points and immune treatments, *D. suzukii* had significantly more plasmatocytes, producing up to five times more plasmatocytes than *D. melanogaster*. *D. suzukii* larvae also produced significantly more podocytes than *D. melanogaster*, including constitutively produced podocytes, which are not normally found in *D. melanogaster* larvae. Furthermore, *D. suzukii* larvae produced significantly more lamellocytes than *D. melanogaster* larvae. We found no effect of immune challenge on plasmatocyte or podocyte numbers, although as expected there were significantly more lamellocytes in immune-challenged flies. Interestingly, the *D. melanogaster* genome strain used in the present study was not resistant to LbG486, unlike *D. suzukii*, (see below), and also showed no significant increase in lamellocyte numbers at two time-points post-infection when infected by LbG486 (Figure 4A, 4B). Finally, there were significant species-by-immune challenge interaction effects on podocyte and lamellocyte numbers in some experiments, usually due to significantly greater induction of these cell types after an immune challenge in *D. suzukii*. Thus, like *D. melanogaster*, *D. suzukii* induces hematopoiesis and/or hemocyte differentiation during a cellular immune response, although this induction is often stronger than that observed in *D. melanogaster*.

**Figure 13:**
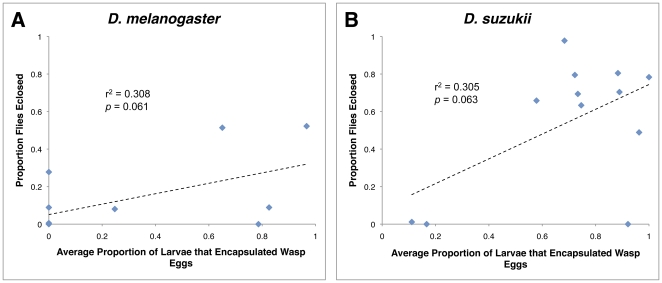
Relationship between encapsulation rate and fly eclosion. Average proportion of fly larvae that encapsulated a wasp egg for *D. melanogaster* (A) and *D. suzukii* (B).

**Figure 14:**
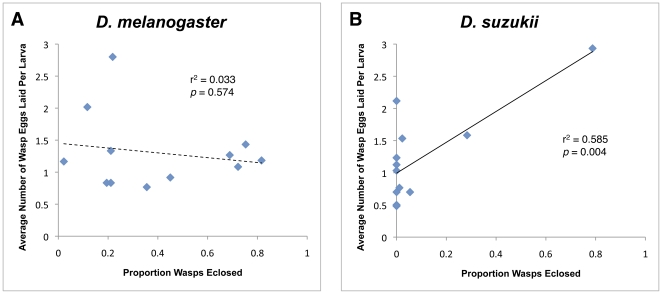
Relationship between wasp eclosion success and number of eggs wasps choose to lay in a host. There was no significant relationship for the panel of wasp species attacking *D. melanogaster* (A), but there was a significant relationship for the panel of wasp species attacking *D. suzukii* (B).

We next tested the effects of fly species and immune challenge on fly crystal cell counts using standard regression methods (Figure 5). There was a significant effect of species on crystal cell numbers in the piercing experiment, whereby *D. suzukii* had more than three times the number of constitutively produced crystal cells compared to *D. melanogaster* (Figure 5B). There was a similar, albeit non-significant trend in the wasp-attack experiment (Figure 5A). There were consistent, significant immune challenge effects of crystal cell counts, whereby both species showed significant reductions in crystal cell numbers following wasp infection or piercing, suggesting either crystal cells or their crystals (which are thought to contain the melanization precursors [36]) were spent during the wound healing or immune responses. Significant melanization was observed around the wound site in both species.

In order to confirm that hemocyte count differences between *D. melanogaster* and *D. suzukii* are general, we conducted further hemocyte counts experiments using two more strains of both fly species (Figure 6). Once again, we found a significant effect of species on constitutive numbers of plasmatocytes, podocoytes, and crystal cells, with the *D. suzukii* strains having greater numbers of these cell types in every case.

### Fly Resistance

In the next experiment, both host species were infected with a panel of parasitoid wasps. Since we did not observe the flies and wasps for the duration of the infection period, it was important to know whether wasp infection rates were similar across the two fly species, so that any difference in fly eclosion could be attributed to a successful encapsulation response rather than a lack of infection. We compared the average number of eggs laid in the larvae of both fly species by the panel of parasitoid wasps. Although significant differences existed in the number of eggs laid by different wasp strains within a fly species (*D. melanogaster* ANOVA *p*<10^−4^, *D. suzukii* ANOVA *p*<10^−4^) (Figure 7), there was no overall difference between fly species in the number of eggs laid by the different wasp species (Figure 8), which averaged close to 1.25 eggs per fly larva in both fly hosts. Thus there was no evidence of an overall infection preference by wasps for one fly species over the other, and no evidence of differences in alternative mechanisms of host defense, such as behavioral or physical immunity (*e.g.*, a thickened cuticle) by the flies.

*D. suzukii* was able to melanotically encapsulate at least a small proportion of eggs from all 21 larval parasitoid wasp strains tested, whereas *D. melanogaster* was able to encapsulate some proportion of eggs from only 8 of 21 wasp strains (LbFr, LbG486, LcNet, G1Fl, G1Haw, AtFr, AtSw, and Aph1Atl) and only 5 of 12 wasp species (Figure 9A). The difference in the proportion of wasp species that the flies could melanotically encapsulate was statistically significant (Fisher's exact test *p* = 0.005). Qualitative melanotic encapsulation differences between *D. melanogaster* and *D. suzukii* held across additional strains tested of both species (Figure 9B). As expected, the *D. suzukii* strains were able to encapsulate 3 of the 4 larval parasites tested (Lb17, GxUg, ApIndo, but not AjJap), while *D. melanogaster* was not able to encapsulate any of the parasites.

*D. suzukii* was also consistently more resistant to our panel of parasitoid wasp species than *D. melanogaster* (Figure 10, 11). A greater proportion of *D. suzukii* eclosed after wasp infection compared to *D. melanogaster* for 20 of the 24 wasp strains tested, the exceptions being *D. suzukii* infected by wasp strains G1Fl and G1Haw (for which no flies of either species eclosed), AjJap, and Tri1Cal. This corresponded to a significantly higher fly eclosion rate for *D. suzukii* compared to *D. melanogaster* across wasp species (Figure 11A). Furthermore, a lesser proportion of wasps eclosed from infected *D. suzukii* larvae compared to *D. melanogaster* for 19 of the 24 wasp strains tested, the exceptions being *D. suzukii* infected by wasp strains G1Fl, G1Haw, AjJap, Tri1Cal, and Pac1Atl. This corresponded to a significantly lower wasp eclosion rate in *D. suzukii* compared to *D. melanogaster* across wasp species (Figure 11B). The proportion of attacks that led to death of both the fly and the wasp growing within the fly was also lower in *D. suzukii*, with *D. suzukii* showing a lower proportion of death than *D. melanogaster* for 17 of the 24 wasp strains tested. However, this difference was not significant across wasp species (Figure 11C). When we tested additional strains of both *D. suzukii* and *D. melanogaster*, we found qualitatively similar eclosion results (Figure 12). As expected, a greater proportion of *D. suzukii* eclosed following infection compared to *D. melanogaster* for 3 wasp strains (Lb17, GxUg, ApIndo) *D. suzukii* was previously successful against, but not for two wasp strains *D. suzukii* previously did poorly against (AjJap, Tri1Cal).

Given our understanding of the Drosophila immune response against wasp parasitism, we expect that flies that successfully encapsulate particular wasp species will also have greater eclosion success against those same wasp species. To test this expectation, we assayed for correlations between encapsulation success and fly eclosion for both flies species infected by the panel of wasp species. Although we found a trend in the expected direction for both fly species, there was no significant correlation in either fly species (Figure 13).

### Wasp Choice

Previous work using *D. melanogaster* has shown that wasps can differentiate between infected and un-infected flies, and that they preferentially lay eggs in fly hosts that have not already been infected [38], [39], [40], [41], [44], [45]. This preference is presumably adaptive because it limits competition between juvenile wasps that require the resources from an entire fly to complete development. Such preference should lead to an under-dispersion of wasp eggs in any group of infected fly larvae, *i.e.*, a more even distribution of eggs per larvae than expected by chance. We found significant under-dispersion of wasp eggs in *D. melanogaster* larvae for 15 of the 21 larval parasite wasp strains (Figure 7). The wasp strains that laid the most eggs in *D. melanogaster* tended to show the least under-dispersion, suggesting that the wasps could not differentiate between infected flies once they were infected with more than one wasp egg [38], [39]. Only 4 of 21 wasp strains showed a significant under-dispersion of eggs across *D. suzukii* larvae. This suggests that whatever cue the wasps use to identify infected *D. melanogaster* larvae, whether it is a tag left by the previous wasp or some aspect of the *D. melanogaster* response to infection [40], is generally missing in *D. suzukii* larvae. In no fly-wasp interaction was there a significant over-dispersion of wasp eggs.

Drosophila parasitoid wasps can also distinguish between fly host species, and preferentially lay eggs in host species in which their offspring have a higher chance of survival [29], [46], [47]. We tested whether larval parasitoid wasps tended to lay more eggs in the fly hosts that their offspring more successfully eclosed from in our trials (note that in our trials the wasps did not have a choice between host species, only whether or not to lay eggs in a single given host) (Figure 14). There was no relationship between wasp species success and the number of eggs laid per larva with *D. melanogaster* as host (r^2^ = 0.033, ANOVA *p* = 0.574). For *D. suzukii*, however, there was a highly significant relationship (r^2^ = 0.585, ANOVA *p* = 0.004) that was due in large part to the wasp species *A. japonica* (strain AjJap) and *Ganaspis sp.1* (combined strains G1Fl and G1Haw). AjJap in particular laid the highest number of eggs in *D. suzukii* in our infection trials, and also had the highest eclosion success.

### Specificity In Fly-Wasp Interactions

As described above, we found significant differences in the number of eggs laid by different wasp strains within fly species but not between fly species. This could mean that wasps that lay higher numbers of eggs in *D. melanogaster* also lay higher numbers of eggs in *D. suzukii*, *i.e.*, some wasps could have generally higher egglay rates than others. However, there was no correlation between the number of eggs laid in *D. melanogaster* and the number of eggs laid in *D. suzukii* for the panel of wasp species (r^2^ = 0.016, ANOVA *p* = 0.696), suggesting that egglay rate is a plastic wasp trait that wasps tailor to the host species they encounter.

There were significant differences across the panel of wasp strains in the infection outcomes within fly species (ANOVA *p*<10^−4^ for all six comparisons: fly survival, wasp survival, death in *D. melanogaster*, *D. suzukii*) (Figure 10). Although these differences in infection outcomes were due to significant variation both between wasp species and within wasp species (variation amongst strains), the largest differences in infection outcomes are seen between wasp species rather than wasp strains. For each fly host, some wasp species were very successful infectors, some were very susceptible to the fly immune responses, and some induced a large amount of death. As described above there were also significant differences in the infection outcomes between fly species. Despite the superior wasp resistance of *D. suzukii*, it is possible that wasps that were more successful in *D. melanogaster* were also more successful in *D. suzukii*, *i.e.*, some wasps are generally more virulent than others. However, there was no correlation in the proportions of any of the three infection outcomes between *D. melanogaster* and *D. suzukii* (fly success r^2^ = 0.102, ANOVA *p* = 0.265; wasp success r^2^ = 0.001, ANOVA *p* = 0.908; death r^2^ = 0.041, ANOVA *p* = 0.489). This indicates there was specificity in the outcome of wasp infections depending on the particular host fly species, despite *D. melanogaster* and *D. suzukii* being part of the same Drosophila species group.

There is a strong influence of wasp phylogeny on *D. melanogaster* infection outcomes. Members of the Leptopilina clade that includes *L. boulardi* and *L. heterotoma* are very successful against *D. melanogaster*, showing an average of 69% wasp eclosion. Infections by *L. clavipes* and members of the genus Ganaspis, which are likewise members of the family Figitidae, did not result in high eclosion rates in *D. melanogaster*, but instead caused an average of 79% death of *D. melanogaster* larvae (Figure 8). Thus, *D. melanogaster* appears to lack an immune mechanism to counter shared virulence strategies of Figitid parasitoids. There appeared to be little influence of wasp phylogeny on the ultimate outcome of *D. suzukii*–wasp interactions, as *D. suzukii* was resistant to the majority of wasps tested. However, the larval parasitoid that eclosed from *D. suzukii* at the greatest rate (79%), *A. japonica*, is endemic to Japan where it is sympatric with *D. suzukii*.

## Discussion

Previous studies have shown that fly species and strains with a greater constitutive production of hemocytes are more resistant against and/or are better able to encapsulate parasitoid wasp eggs [12], [18], [19], [48]. Although a correlation does not necessarily imply causation, these data suggest that evolution of higher constitutive production of hemocytes is a relatively simple way for hosts to defeat one of their most common classes of parasites. However, the previous studies were limited to flies in the melanogaster subgroup and to a few wasp species/strains that represent only a small fraction of the diverse virulence strategies used by Drosophila parasitoid wasps. To determine if increased hemocyte production by flies is a panacea against wasp infection, we first compared hemocyte numbers between *D. melanogaster* and *D. suzukii*, a relative of *D. melanogaster* outside the melanogaster subgroup.

We found that third instar *D. suzukii* larvae made constitutively greater numbers of plasmatocytes, podocytes, and crystal cells than *D. melanogaster* larvae, and also induce greater production of podocytes and lamellocytes (Figure 4, 5, 6). Compared to our recently wild-derived *D. suzukii* strains, the *D. melanogaster* genome strain we used may have had relatively poor genetic immune ability for its species due to its homozyosity and its long-term selection in a lab environment. However, hemocyte counts from the two additional wild-caught *D. melanogaster* strains we assayed were very similar to those from the genome strain. The hemocyte numbers we observed in our *D. melanogaster* strains were also similar to those seen in a variety of other studies where the unit of measurement was cells per larva [33], [35], [42], and also appeared similar to numbers found in studies that counted cells per volume of hemolymph (using a rough conversion factor of approximately 0.5 uL hemolymph per third instar larva) [12], [19], [43], [49], [50]. Thus, we have no reason to believe that differences we observe between our *D. melanogaster* and *D. suzukii* strains were due to a biased sampling of strains rather than actual species differences. In comparison with hemocyte numbers from other studies, *D. suzukii* appears to have somewhat greater constitutive hemocyte counts than *D. simulans*, which has the highest counts of any member of the melanogaster subgroup [12].

Using a diverse panel of parasitoid wasp strains and species, we found that infection rates in *D. melanogaster* and *D. suzukii* were similar (Figure 7, 8), but that *D. suzukii* was significantly better at melanotically encapsulating, and surviving infection by, the wasps (Figure 9, 10, 11,12). The panel of wasps included relatively specialist and generalist wasp species, such as *L. boulardi* and *L. heterotoma*, respectively [15], as well as relatively immune evasive versus immune suppressive wasp species, such as *A. tabida* and *G. xanthopoda*, respectively [51], [52]. Our data suggest that a general protection against parasitoid wasps is afforded to fly species that have higher constitutive hemocyte loads. The association between hemocyte load and encapsulation ability reported previously [12] also appears to extend beyond the melanogaster subgroup of fly hosts, as *D. suzukii* is part of the melanogaster group but not the melanogaster subgroup. Future infection trials using the same panel of parasitoid wasps, but a much wider range of fly species, will be needed for determining the true extent of the relationship between hemocyte load and resistance against parasitoid wasps.

The current model for the melanotic encapsulation process is that plasmatocytes act as sentinels of wasp infection and signal to activate other circulating plasmatocytes as well as the lymph gland once infection is recognized [4], [5]. The activated plasmatocytes develop cytoskeletal projections and become known as podocytes, which may be an intermediate form between the smaller plasmatocytes and larger lamellocytes [6], [7]. Lamellocytes are also induced via differentiation of pro-hemocytes in the lymph gland. The lamellocytes then migrate towards and surround the wasp egg, forming a tight capsule. The capsule becomes melanized, but it is not yet known whether melanin precursors stored in crystal cells are used in this process. Thus, any or all of the hemocyte cell types that *D. suzukii* produced in excess may have been responsible for the relatively high resistance of *D. suzukii* against wasp eggs.

Flies with more hemocytes may suffer fewer effects of wasp venom for a variety of reasons, enabling them to mount a quicker and/or stronger encapsulation reaction against wasps. For example, venoms often alter hemocyte structure and function [50], [53], and thus an increased number of hemocytes could potentially dilute the effects of a standard dose of venom. Alternatively, hemocytes may be responsible for destroying venom components found in the hemolymph, via endocytosis or some other mechanism, preventing the venom from exerting its effects on other tissues. It is unclear whether an excess of constitutively produced hemocytes (plasmatocytes, crystal cells) or the increased induced production of podocytes and lamellocytes drives the relationship between hemocyte counts and wasp resistance, but the distinction may be unimportant given that constitutively produced cells can differentiate into induced cell types [6], [7]. However, in support of the idea that constitutive production of hemocytes alone is not sufficient for wasp resistance, Drosophila species of the obscura group that make relatively high numbers of plasmatocytes, but apparently do not produce a lamellocyte class of cells, are unable to encapsulate foreign objects and are highly susceptible to wasp infection [54], [55].

Unlike for *D. melanogaster*, larval parasitoid wasps rarely under-dispersed their eggs across *D. suzukii* larvae. Wasps are thought to discriminate naïve host larvae from previously infected larvae either by recognizing a mark left by the previous wasp, or by recognizing the host response to infection [40]. Given that *D. suzukii* has a significantly more robust immune response against wasp infection than *D. melanogaster*, it seems unlikely that these wasps use host immune cues to avoid superparasitism. If fly hemocytes are responsible for clearing wasp venom components from the hemolymph, wasp “possession marks” might also be lost in fly hosts that make abundant hemocytes, leading to more random dispersal of wasp eggs across host larvae.

We expected to find a correlation between encapsulation ability and fly success in both *D. melanogaster* and *D. suzukii*, but although there was a trend in this direction, the correlations were not significant (Figure 13). Three factors likely contribute to this lack of correlation. First, we counted fly larvae as having successful encapsulations if any encapsulation was seen, even if flies were super-parasitized and hadn't encapsulated all wasp eggs they were infected by. Thus, flies scored as showing encapsulation could still succumb to infection. Second, some fly-wasp combinations that yielded encapsulations culminated in neither fly nor wasp eclosion, but high rates of death of by both fly and wasp. Third, wasp parasites sometimes die inside their fly hosts even if the fly has not encapsulated them by the time-point we assayed.

Interestingly, *D. suzukii* does not have a clear survival advantage over *D. melanogaster* when infected by the two pupal parasite species (three strains) in our panel of wasps. Very little is known about the determinants of infection outcomes with regards to pupal parasites of flies, or even whether venom plays an important role. Although Trichopria acts as a pupal endoparasitoid, the Drosophila pupal stage does not appear able to mount melanotic encapsulation responses against them. Furthermore, Pachycrepoideus lays its eggs in the space between the pupal case and the pupa, and acts as an ectoparasite for most of its development [29], which could negate any ability the flies have to mount an internal, physiological immune response. In other systems, pupal parasitoid wasps are known to have more generalist host ranges than larval parasites [30], [31], but they do not have unlimited host ranges either, so some specificity in their utilization of host resources is inherent. Although our data suggests increased hemocyte load has little effect on fly resistance against pupal parasites, a definitive statement will require data from a greater range of pupal parasite species.

Still, if increased hemocyte load provides general protection against larval parasitoids, why do some fly species, such as *D. melanogaster*, produce such low numbers compared to their close relatives? Hosts face an evolutionary tradeoff between investing in immune responses against parasites versus investing in other aspects of fitness [56], [57], [58], [59]. The constitutive production and maintenance of hemocytes must obviously impart an energetic cost on the host, diverting resources from other aspects of host fitness. Thus, if hosts are rarely infected by wasps in nature, or are commonly infected by specialist wasps that can overcome hemocyte-based immunity, it may make evolutionary sense to invest in fecundity rather than immunity, or in other aspects of immunity, such as behavioral immunity. On the other hand, investment in high constitutive hemocyte levels might be selected in host species that are commonly infected by non-specialist parasites.

Although *D. suzukii* is generally more resistant against larval wasp parasites than *D. melanogaster*, there were a small number of obvious exceptions. *A. japonica* is sympatric with *D. suzukii* in its native east Asian range, and was significantly more successful at infecting *D. suzukii* than *D. melanogaster*. Previous studies showed *A. japonica* successfully parasitizes *D. suzukii* both in the field and in the lab [60], [61]. *A japonica* also laid approximately three times more eggs in *D. suzukii* than in *D. melanogaster*, and laid the highest number of eggs in *D. suzukii* of any larval parasitoid wasp. Altogether, these data suggest *A. japonica* may have co-evolved a specialized virulence strategy able to overcome the high hemocyte load of *D. suzukii*, and may have evolved an infection preference for *D. suzukii* as well. The only other larval parasite able to eclose from *D. suzukii* hosts at any appreciable rate is *Ganaspis sp.1*, an undescribed species collected in Florida and Hawaii. Although *G. xanthopoda* was found to emerge from *D. suzukii* pupae collected in the field in Japan [61], the two *G. xanthopoda* strains used in this study, from Hawaii and Uganda, were very poor infectors of *D. suzukii*, suggesting populations of this wasp species may have locally adapted to *D. suzukii* host use in Japan.

*D. suzukii* has recently spread into Europe and North America as a pest species [20], [21], [22], [23]. It was first documented in the United States in California in 2008, from where it quickly spread to Oregon and Washington. In these west coast states, *D. suzukii* was responsible for up to 80% yield losses in berry and cherry crops depending on location, and is estimated to be causing yearly monetary losses in the range of 500 million dollars [62], [63]. In 2009, *D. suzukii* became established in Florida, and in 2010 reports of collections were made from a handful of new states [23]. However, experimental studies testing the efficacy of various management strategies for *D. suzukii* are as yet lacking [64], [65].

One common pest management strategy is the use of biocontrol agents such as natural enemies (parasites, predators) [66], and parasitoid wasps have successfully controlled numerous other arthropod pests in the past [24], [25], [26], [27], [28]. Furthermore, Drosophila parasitoid wasps often infect a large proportion of fly larvae in natural populations [1], [2], [3], and the potential for an endemic Figitid species (*L. boulardi*) to control native Drosophila populations in California was previously considered [67]. It appears the wasp species with the highest potential for use in biocontrol of *D. suzukii* are the larval parasites *A. japonica* and *Ganaspis sp.1*, and the pupal parasite *Trichopria sp.1*. *A. citri* might also be considered a potential biocontrol agent for *D. suzukii* because of the high death rates it caused in *D. suzukii*, but this wasp had much higher eclosion rates using *D. melanogaster* as host than *D. suzukii*. Because infection trial conditions in this study were designed to be ideal for success of the wasps, and such conditions (easy access to hosts, no competition with other parasites, controlled temperature, abundant resources, etc) are unlikely to be replicated in the field, extensive field experiments will be required to assess the efficacy of the use of parasitoid wasps in *D. suzukii* biocontrol in practice.
